# Molecular characterization of indigenous human adenovirus (HAdV) isolate from healthy infant stool sample and screening of its antibodies in archival serum samples in Türkiye

**DOI:** 10.1371/journal.pone.0328556

**Published:** 2025-07-18

**Authors:** Zafer Yazici, Huseyin Baskin, Seda Gozel, Hanne Nur Kurucay, Cuneyt Tamer, Hamza Kadi, Emre Ozan, Bahadir Muftuoglu, Vahide Bayrakal, Harun Albayrak, Semra Okur-Gumusova, Ahmed Eisa Elhag

**Affiliations:** 1 Department of Virology, Faculty of Veterinary Medicine, Ondokuz Mayis University, Samsun, Türkiye; 2 Department of Medical Microbiology, Faculty of Medicine, Dokuz Eylul University, Izmir, Türkiye,; 3 Department of Quality Improvement in Healthcare and Accreditation, Institute of Health Sciences, Dokuz Eylul University, Izmir, Türkiye; 4 Laboratory of Forensic Microbiology and Biological Defense, Dokuz Eylül University Hospital, Izmir, Türkiye; 5 Department of Virology, Samsun Veterinary Control Institute, Ministry of Agriculture and Forestry, Samsun, Türkiye; 6 Department of Experimental Animals, Faculty of Veterinary Medicine, Ondokuz Mayis University, Samsun, Türkiye; 7 Institute of Biochemistry and Biophysics, Polish Academy of Sciences, Warsaw, Poland; 8 Department of Preventive Medicine and Clinical Sciences, Faculty of Veterinary Sciences, University of Gadarif, Al Qadarif, Sudan; Defense Threat Reduction Agency, UNITED STATES OF AMERICA

## Abstract

Human adenoviruses (HAdV) are significant etiological agents of infections affecting the respiratory, gastrointestinal, urinary and ocular systems, particularly in adults, infants, and immunocompromised individuals. This study presents the molecular identification of a local HAdV strain for the first time from the stool of a healthy infant in Türkiye, isolated in 2003 and stored for two decades in liquid nitrogen. Molecular characterization of this strain was performed, identifying it as HAdV-C6. Phylogenetic analysis revealed high nucleotide identity (97%) with global strains from Russia, China, Japan, and the USA. A serum neutralization test was conducted to determine the current circulation of this strain, indicating a 9.5% seropositivity rate in archival serum samples collected for the West Nile virus surveillance project. This study provides insights into the persistence and molecular epidemiology of HAdV strains circulating in Türkiye, highlighting the need for continuous surveillance and whole-genome sequencing to assess potential recombination events and genetic variations.

## 1. Introduction

Human adenoviruses (HAdV), also known as human mastadenoviruses, are members of the *Mastadenovirus* genus in the *Adenoviridae* family, featuring a linear double-stranded DNA genome [1,2]. Since their discovery in 1953, more than 110 HAdV types have been identified and classified into seven species (A-G) based on genetic characteristics, tissue tropism, and clinical manifestations [3,4]. The vast majority of these HADV types (57) have been placed among the HAdV-D species [3,5]. Most of the adenoviruses nowadays have been implicated in a wide range of infections, including respiratory illnesses, gastroenteritis, conjunctivitis, and systemic infections in immunocompromised individuals, and are recognized as one of the essential etiological agents for acute diarrhea-diagnosed patients, particularly for human adenoviruses (HAdV) [3,5]. HAdV infections are most common in children under five years of age and are known to cause outbreaks, particularly in confined environments such as schools and military barracks [6].

Among all aforementioned HAdV species, notably, types 40 and 41, belonging to HAdV-F, are the main causative agents of gastroenteric illness in humans [3,5]. Although HAdV-A, B, C and D are responsible for respiratory illness, conjunctivitis as well as systemic infections, they can also be associated with acute gastroenteritis [4]. Among the seven species, C (HAdV-C) is known for causing respiratory diseases, especially in infants and young children [7]. HAdV-C types, such as HAdV-1, HAdV-2, HAdV-5, and HAdV-6, are commonly isolated from clinical samples and environmental reservoirs, including stool and sewage [8]. Notably, HAdV infections often present asymptomatically in healthy individuals, but they can become reactivated in immunocompromised patients, leading to more severe outcomes [9].

Türkiye has actively participated in global poliomyelitis eradication programs, resulting in increased screening of stool samples for enteroviruses, with the added benefit of detecting other viruses like adenoviruses [10]. Despite extensive surveillance and advances in our understanding of HAdV pathogenesis, limited data exist on the molecular epidemiology of HAdVs and their circulating strains in Türkiye. The identification and molecular characterization of local HAdV strains are crucial for understanding their circulation, transmission dynamics, and potential role in outbreaks [11].

Previous studies have shown that HAdV-C6, though rare, is a globally distributed strain isolated from respiratory and stool samples across continents [12]. However, no detailed studies have been conducted on HAdV-C6 isolates in Türkiye. This study, therefore, aims to fill this gap by providing the first molecular identification and phylogenetic analysis of an indigenous HAdV-C6 strain isolated from an infant stool sample collected during a national poliomyelitis surveillance program in 2003. To the best of our knowledge, this was the first HAdV isolation propagated in cell culture in Türkiye 20 years ago. At the end of the long-term storage, this indigenous isolate is expected to provide information about human adenoviruses circulating in Türkiye in the past because we additionally aimed to screen human serum samples collected for West Nile virus surveillance in 2020 to assess the presence of neutralizing antibodies against this local strain, providing insight into the long-term circulation of HAdV in Türkiye.

## 2. Materials and methods

### 2.1. Origin of the local isolate and serum samples

In 2003, a local HAdV was isolated in cell culture from a stool sample from a healthy infant collected for the national poliomyelitis eradication program of Türkiye. The isolate was initially confirmed via latex agglutination and direct fluorescence technique and archived [13]. Since the long-term storage of this local isolate in liquid nitrogen (−195ºC) began over 20 years ago, no molecular identification or characterization has been conducted until the present study.

Additionally, 400 human serum samples collected during West Nile virus surveillance in 2020 [14] were screened for HadV-specific antibodies to determine whether this local isolate was still circulating.

### 2.2. Ethics statement

The serum samples used in this study were obtained from archival stocks previously used in our earlier study [14], for which ethical approval was granted by the Ondokuz Mayis University Ethical Committee for Clinical Trials (Approval No. 08/1523–163; Date: 15/05/2018), and formal informed consent was obtained. The authors had access to information that could identify individual participants during or after data collection. No additional ethical approval was required, as all experimental procedures were conducted using cell culture techniques, and no animal models were involved in this research. Also, the virus used was retrieved from our laboratory stock and originally isolated from an archived stool sample collected over 20 years ago, with the associated data previously published [13].

### 2.3. Cell line and virus

HEp-2 (Human laryngeal epithelial carcinoma cell line) cell line, provided from the cell and virus collection of the Department of Virology at the Faculty of Veterinary Medicine in Ondokuz Mayis University, Samsun, Türkiye, was used in this study for virus recultivation. Cells were grown in Eagle’s Minimum Essential Medium (EMEM, Sartorius, UK) supplemented with 5% fetal calf sera (FCS, Sigma UK) and 1% antibiotics and were also incubated at 37ºC with 5% CO_2_.

For recultivation of local human adenovirus (HAdV), the local isolate was inoculated into freshly prepared HEp-2 cells using a conventional method. Infected cell cultures were monitored daily by an inverted microscope (Olympus, Japan) until the cytopathic effect (CPE) reached 80−100%. After that, infected cells were frozen and thawed three times and stored at −20ºC until further use as 1.5 ml aliquots.

### 2.4. 50% tissue culture infective Dose (TCID_50_) assay

Local HAdV isolate was diluted tenfold in DMEM supplemented with 2% FCS. 100 µl of each dilution was put into quadruplicate in 96 well plates. Then 50 μl of 1.0 × 10^5^ HEp-2 cells were added to each well, and the plates were maintained at 37°C for 72 hours within a humidified incubator with 5% CO_2_. After incubation, CPE was checked and the 50% tissue culture infective dose was calculated by the method of Reed & Muench [15] and expressed as log_10_ TCID_50_/ml.

### 2.5. Nucleic acid extraction and polymerase chain reaction (PCR)

Viral DNA was extracted from HAdV-infected cells using High Pure Nucleic Acid Kit (Roche, Germany) according to the manufacturer’s instructions. The confirmatory PCR test targeted the conserved region of the hexon gene of HadV using forward (AD1:5′-CTG ATG TAC TAC AAC AGC ACT GGC AAC ATG GG-3′) and reverse (AD2:5′-GCG TTG CGG TGG TGG TTA AAT GGG TTT ACG TTG TCC AT-3′) primers as previously described [16,17].

### 2.6. CPE-based serum microneutralization test (SNT)

A 1:5 dilution of each human serum sample was made in duplicate in 96 well plates using 50 μl of each test serum diluted in DMEM without FCS. 100 TCID_50_ of the local isolate was then added to the corresponding wells and incubated at 37°C for 1 h. Finally, 5.0 × 10^4^ HEp-2 cells were added to each well, and the plate was incubated at 37°C for 72 h in a humidified incubator with 5% CO_2_. The results were evaluated based on the appearance of CPE, which was observed under an inverted microscope on day 3 of infection. No CPE seen in duplicated wells indicated the existence of antibodies against the virus, so the result was evaluated as SNT positive or vice versa.

### 2.7. Sequencing and phylogenetic analysis

The PCR product was purified using a QIAquick PCR purification kit (Qiagen, Hilden, Germany), according to the manufacturer’s instructions. Then, the amplicon was sequenced using the Sanger method by The Black Sea Advanced Technology Research and Application Center (KITAM) of the Ondokuz Mayis University, Samsun, Türkiye.

The nucleotide sequence was assembled with BioEdit ver 7.2.5 [18] and compared to the GenBank database. The dataset for phylogenetic analyses was constructed from GenBank references of HAdV A-G types, vaccine strains, and particularly sequences of HAdV C type (Table 1). MEGA 11 ver 11.0.13 [19] was used for the best model selection and phylogenetic tree construction. The phylogenetic tree was constructed using maximum likelihood (GTR + G + I substitution model) with evaluation through 1000 bootstrap replicates.

**Table 1:** The information on strains selected for use in phylogenetic analysis for the partial sequence of the L3 gene (hexon protein) of HAdV.

Order	GenBank No	Species	Type	Strain	Country	Year
1	NC_001460	HAdV A	12	Huie	NA	NA
2	NC_011202	HAdV B	11	Slobitski	NA	NA
3	NC_011203	HAdV B	3	GB	NA	NA
4	MN936178*	HAdV B	7	Vaccine	USA	2008
5	AY594256*	HAdV B	7	HAdV-7vac	USA	NA
6	AY495969*	HAdV B	7	Vaccine strain	China	NA
7	AF065067*	HAdV B	7	55142	NA	NA
8	NC_001405	HAdV C	2	NA	NA	NA
9	AC_000007	HAdV C	2	NA	NA	NA
10	AC_000008	HAdV C	5	NA	NA	NA
11	AC_000017	HAdV C	1	NA	NA	NA
12	FJ349096	HAdV C	6	Tonsil 99 prototype	USA	1953
13	MH322392	HAdV C	6	human/Shanxi-CHN/180/2009	China	2009
14	LC068720	HAdV C	6	1050158	Japan	2005
15	JX423389	HAdV C	6	human/USA/ak31_AdV6/2007/6[P6H6F6]	USA	2007
16	KU923804	HAdV C	6	19/N.Nov/RU/2015	Russia	2015
17	HQ413315	HAdV C	6	ATCC VR-6	USA	1953
18	HQ003817	HAdV C	57	human/RUS/16700/2001/57[P1H57F6]	Russia	1997
19	MH121114	HAdV C	89	47C2	Germany	2017
20	OR777161	HAdV C	108	HAdV-C108/USA/2D9/2009	USA	2009
21	NC_012959	HAdV D	54	Kobe_H	Japan	2000
22	NC_010956	HAdV D	9	Hicks; NIAID V-209-003-014	NA	NA
23	NC_003266*	HAdV E	4	vaccine (CL 68578)	NA	NA
24	AY594254*	HAdV E	4	Vaccine strain	NA	NA
25	MN936177*	HAdV E	4	vaccine	USA	2008
26	NC_001454	HAdV F	40	Dugan	NA	NA
27	DQ923122	HAdV G	52	T03-2244	USA	2003
28	PP500720.1**	HAdV C	6	ZYHB05TR	Türkiye	2005

## 3. Results

**Fig 1:**
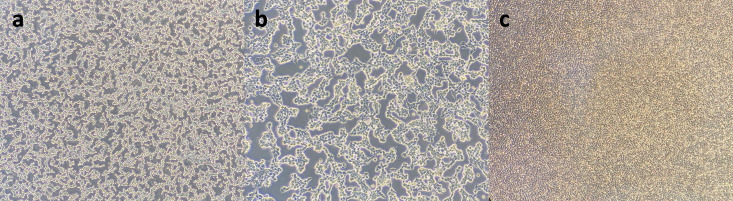
Cytopathic effect (CPE) images were obtained from the third passage of the HAdV-C6 isolate in the HEp-2 cell line at 24h post-infection using an inverted microscope. A and B: 10x and 40x magnifications of HAdV-C6 CPEs; C: cell control (10x).

**Fig 2:**
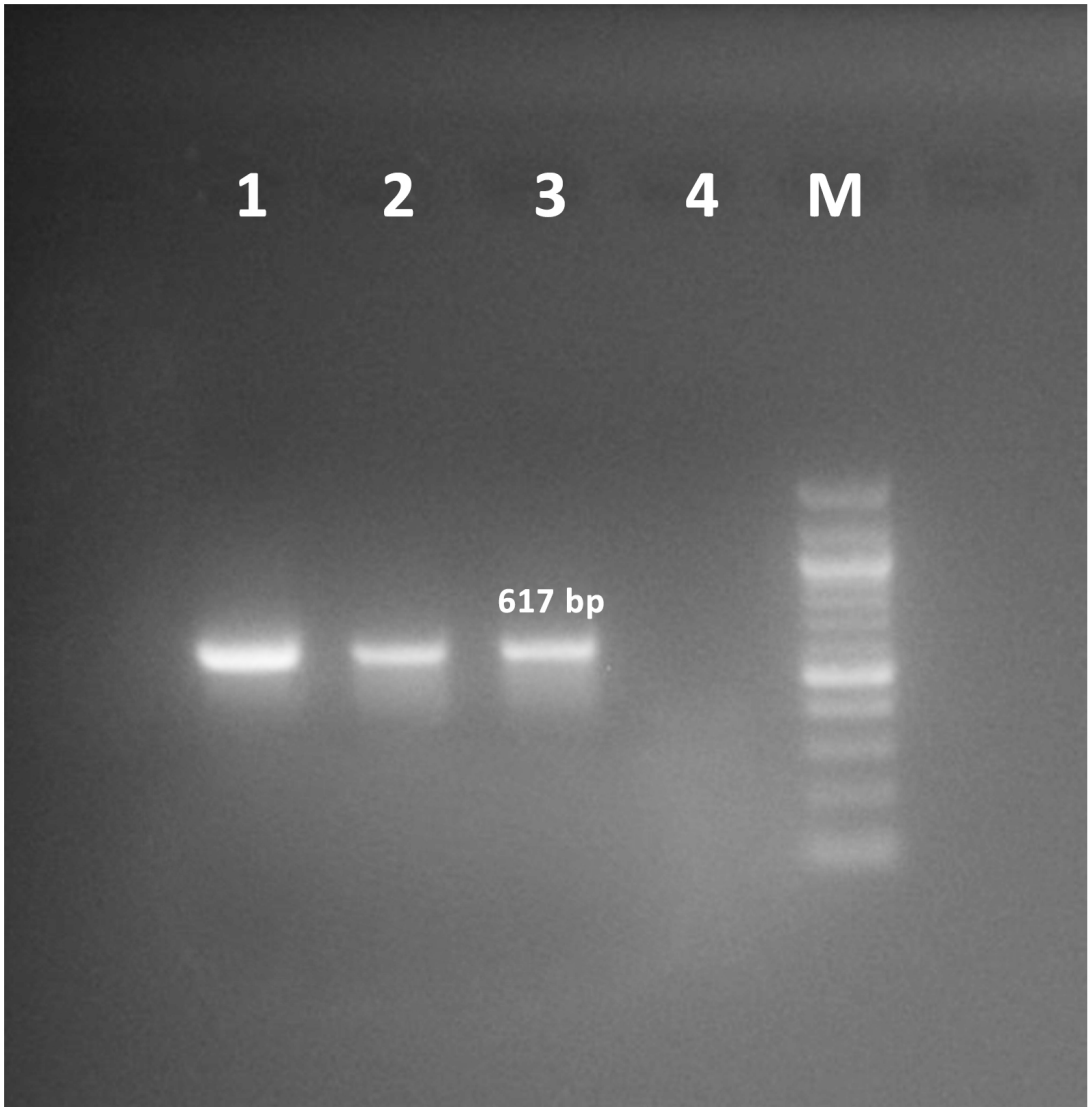
An agarose gel electrophoresis image displaying HAdV PCR products. Lane 1-2: Third passages of HAdV-C6 isolates; Lane 3, 4, and M: positive control, negative control, and 100 bp Ladder, respectively. A 617 bp fragment indicating the hexon gene of HAdV which were obtained at the end of PCR amplification.

The local HAdV isolate was cultivated in the HEp-2 cell and grew up to three serial passages. At the end of the third passage, over 80% of typical adenovirus CPE features, including webbing or lacy appearance and rounding of cells, besides detachment from the flask surface, were observed after 72 h. (**Fig 1**). Also, PCR amplification of the hexon gene confirmed the presence of HAdV by yielding a distinct 617 bp product from supernatants of the third passage of our isolate (**Fig 2**).

TCID_50_ value of the local isolate was calculated to be 6.85 x 10^-5^/mL.

The serum microneutralization test was applied to serum samples that were collected from humans for WNV surveillance to investigate whether the local HAdV isolate was still in circulation. Of 400 sera, 38 were found to be positive for the local HAdV isolate in terms of neutralizing antibodies, indicating overall seropositivity rate of 9.5%. At the end of the sequencing stage, the local HAdV isolate was coded as ZYHB05TR and submitted to GenBank with the accession number PP500720.1. Sequencing results revealed that our local HAdV belonged to genotype C. The 27 strains of human adenovirus seen in **Table 1** were selected from GenBank and compared with our local strain, ZYHB05TR (PP500720.1), in terms of nucleotide identity and amino acid similarity.

As seen in Table 2, our strain shared high nucleotide identity (>97%) with HAdV strains from China (MH322392), Japan (LC068720), the USA (JX423389, HQ413315, FJ349096) and Russia (KU923804), suggesting close genetic relationships. In contrast, the reference strains NC001405, AC_000007, AC_000008, and AC_000017 had < 90% nucleotide identity and amino acid similarity with this indigenous strain. We also detected 95.42% amino acid similarity for the local isolate with the above-mentioned strains that had > 97% nucleotide identity.

**Table 2:** Amino acid and nucleotide similarities according to the partial sequence of L3 gene (hexon protein) of HAdV C.

	GenBank No	Type	Amino acid similarities (%)													
			NC_001405	PP500720.1*	AC_000007	AC_000008	AC_000017	FJ349096	MH322392	LC068720	JX423389	KU923804	HQ413315	HQ003817	MH121114	OR777161
**Nucleotide similarities (%)**	**NC_001405**	2		87.58	100.0	82.55	89.33	91.50	91.50	91.50	91.50	91.50	91.50	94.70	98.70	98.70
	PP500720.1*	6	88.67		87.58	79.19	82.67	95.42	95.42	95.42	95.42	95.42	95.42	88.74	88.24	88.24
	**AC_000007**	2	100.0	86.7		82.55	89.33	91.50	91.50	91.50	91.50	91.50	91.50	94.70	98.70	98.70
	**AC_000008**	5	77.33	76.44	77.33		80.54	83.22	83.22	83.22	83.22	83.22	83.22	82.43	82.55	82.55
	**AC_000017**	1	84.44	80.89	84.44	74.61		85.33	85.33	85.33	85.33	85.33	85.33	87.84	89.33	89.33
	FJ349096	6	89.32	97.60	89.32	78.00	81.78		100.0	100.0	100.0	100.0	100.0	93.38	92.16	92.16
	MH322392	6	89.11	97.82	89.11	78.00	81.56	99.78		100.0	100.0	100.0	100.0	93.38	92.16	92.16
	LC068720	6	89.11	97.82	89.11	78.00	81.56	99.78	100.0		100.0	100.0	100.0	93.38	92.16	92.16
	JX423389	6	89.11	97.82	89.11	78.00	81.56	99,78	100.0	100.0		100.0	100.0	93,38	92.16	92.16
	KU923804	6	89.32	97.60	89.32	78.22	81.56	99.56	99.78	99.78	99.78		100.0	93.38	92.16	92.16
	HQ413315	6	89.32	97.60	89.32	78.00	81.78	100.0	99.78	99.78	99.78	99.56		93.38	92.16	92.16
	HQ003817	57	90.51	87.20	90.51	75.56	82.89	89.18	88.96	88.96	88.96	88.74	89.18		94.70	94.70
	MH121114	89	98.05	88.02	98.05	76.67	84.00	88.67	88.45	88.45	88.45	88.67	88.67	88.96		100.0
	OR777161	108	98.05	88.02	98.05	76.67	84.00	88.67	88.45	88.45	88.45	88.67	88.67	88.96	100.0	

The NCBI reference isolates are shown in bold.

The isolate obtained in this study is shown in bold and asterisks (*).

**Fig 3:**
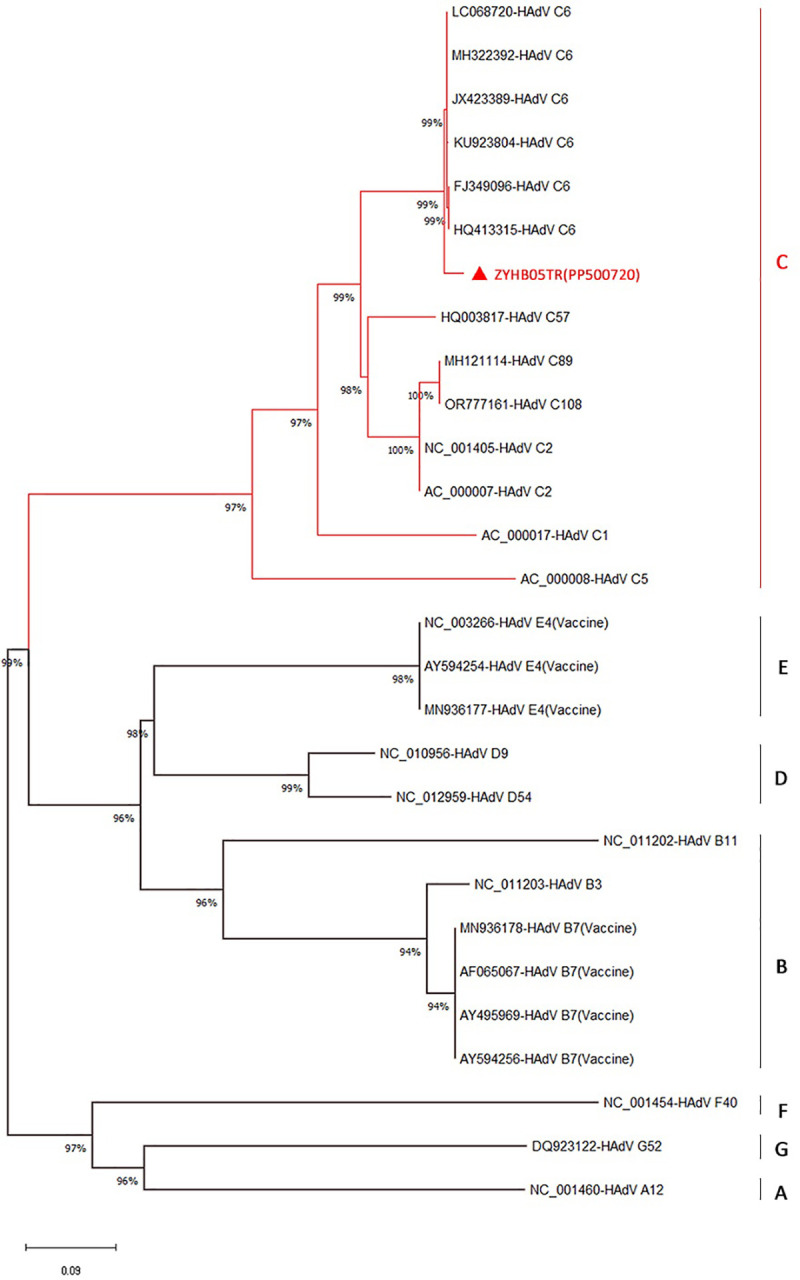
The phylogenetic analysis of the HAdV strain isolated in this study. The tree was constructed from a partial analysis of the L3 gene (hexon protein) of HadV using the maximum likelihood method with MEGA11 software. The robustness branching pattern was tested with 1000 bootstrap replications. According to the phylogenetic tree, the current strain was in genotype C (marked in red and with a ▲). The HAdV sequences were named using their GenBank accession number, strain name, and genotype.

Through sequence analysis, we obtained a 459 nt (153 aa) sequence from the L3 gene region encoding the hexon protein of our isolate, ZYHB05TR (PP500720.1), which was subsequently utilized in phylogenetic analyses. Initially, NCBI Nucleotide BLAST and Protein BLAST analyses were conducted, revealing significant similarities with HAdV-C6 isolates. Subsequently, a dataset for phylogenetic analysis was compiled, comprising the above 27 isolates (representing 16 different serotypes) from seven known HAdV species A-G, including our study sample. Consistent with the findings of the BLAST analyses, our ZYHB05TR isolate clustered closely with HAdV C6 isolates in the phylogenetic analysis. The ZYHB05TR isolate exhibited a high degree of nucleotide identity (97.60–97.82%) and amino acid similarity (95.42%) with HAdV C6 sequences (Fig 3). However, lower similarity rates were observed with other serotypes of HAdV C, with the lowest nucleotide and amino acid similarities observed with HAdV C5 (GenBank No: AC_000008), at 76.44% and 79.19%, respectively. We identified seven amino acid mutations between our ZYHB05TR (PP500720.1), isolate and selected HAdV C6 isolates within the 386th and 538th amino acid positions of the hexon protein (D454N, E459K, R481K, D494N, N510T, D513N, and D526N). Interestingly, while the six HAdV C6 isolates selected for phylogenetic analysis shared identical amino acid residues at these mutation sites, our study isolate, ZYHB05TR (PP500720.1), exhibited distinct residues.

## 4. Discussion

Human adenoviruses are one of the viral families that are considered as a common cause of multi-systemic illnesses, with clinical manifestations including pneumonia involving both upper and lower respiratory tract infections (HAdV-B, C, and E), gastroenteritis (HAdV-A, F, and G), keratoconjunctivitis (HAdV-B and D), hepatitis (HAdV-C), encephalitis (HAdV-A, B, and D) and cystitis (HAdV-B) [20]. Although they are highly contagious and can easily spread via air droplets, close contact, and fecal-oral routes, sometimes, it is not reported [21]. HAdVs can infect people from all age groups, but infants, the elderly, and immunocompromised patients are more sensitive than others, particularly to HAdV-C, which is one of the common species in many countries [22].

HAdV-B, C, D, E and F in Türkiye has been reported in previous studies [23,24]. While HAdV-B and C were also shown with a 43.5% ratio as predominant strains in these studies, the vast majority were obtained from respiratory and ocular system specimens and characterization from stool samples, which remains limited [23]. Although the HAdV-C genotype includes non-enteric adenovirus types, a high volume of the virus can be excreted through stools [25]. At the same time, some international studies reported the isolation of HAdV–C from stools, such as C57 type in Azerbaijan, C12, C5 and C6 in Gabon [25,26]. Furthermore, locally, limited studies in Türkiye have reported the isolation of different HAdV species and types from stool specimens without molecular typing until now [13,27]. For instance, we have isolated this HAdV strain from the stool of healthy children during the surveillance program for acute flaccid paralysis in Türkiye in 2003, but molecular typing could not be carried out at that time [13]. In the current study, after gathering resources, we molecularly characterized this local HAdV strain stored at −195°C to complement the lack of study and continue research efforts. We did not encounter any problems with this isolate when it was inoculated into cell culture and revived after 2 decades. It grew quickly and showed full CPE characteristics, as expected.

Sequencing and phylogenetic analysis indicated that the detected HAdV-C6, one of the HAdV genotype C members, was consistent with the previous studies. These results could be interpreted as normal. HAdV-C genotypes are widespread across the world and are of 8 serotypes (C1, C2, C5, C6, C57, C89, C104, and C108) [24]. They are responsible for respiratory tract infections, commonly in children, and viruses are shed through air droplets and close contact with feces [28].

The current study provides significant insights into the molecular characterization of HAdV strains circulating in Türkiye. The isolation of an indigenous HAdV-C6 strain from the stool of a healthy infant highlights the persistence and silent circulation of adenoviruses in the population. Additionally, a serum neutralization test was conducted to determine whether the local strain was in circulation or not. We determined 9.5% seropositivity rate against our local HAdV-C6 serotype. This result indicates that our local isolate is still in circulation among the Turkish population. In previous studies, HAdV-C6 seroprevalence has been reported as low type with rates of approximately 8.5% among adults and 2% among children in the United States, and 12% among healthy adults in China. However, higher seroprevalence rates have been also observed, reaching 78% in Thailand and Cameroon, 73% in South Africa, and 71% in Malawi [29]. Our observed seropositivity rate aligns closely with the lower end of this spectrum and appears to be comparable to rates reported from the United States and China, suggesting a persistently moderate circulation of HAdV‐C6 in Türkiye. Furthermore, the clinical relevance of these antibodies remains unclear. While the presence of IgG antibodies indicates prior exposure, it does not necessarily correlate with protection against reinfection or disease severity, as HAdVs can undergo genomic recombination and generate novel strains capable of evading host immunity [11]. In addition, longitudinal studies could help assess the long-term immunity conferred by HAdV-specific antibodies and their role in protecting against reinfection or severe disease.

The molecular characterization and phylogenetic analysis, based on the hexon gene sequence, showed high nucleotide identity with strains from other regions such as Russia, China, and the USA. This observation aligns with previous studies demonstrating the global dissemination and genetic conservation of HAdV-C6 strains across diverse geographical locations [12,30]. Our isolate clustered with Russian, USA, Japanese and Chinese strains and showed over 95% nucleotide identity with them. Moreover, the identification of distinct amino acid substitutions for our isolate provides novel insight into local viral evolution. Previous studies reported HAdV-C6 to be prevalent in Russia and the closest countries like Japan and China [21]. Türkiye is also one of the closest countries to Russia. It is possible that strong international trade and tourism affairs among both countries since the 1990s could pave the way for this genotype entering Türkiye, which helps to understand the close phylogenetic relationship between the Turkish HAdV-C6 isolate and those from Russia and China [1,15].

HAdVs are known to be highly stable in the environment and can survive in sewage and water systems for extended periods, which increases their potential for global spread through fecal-oral transmission [31]. The presence of such a genetically conserved strain in Türkiye, two decades after its initial isolation, underscores the need for continuous surveillance and monitoring of adenoviruses in both clinical and environmental samples [16].

Given the ubiquity and genetic diversity of HAdVs, continuous surveillance is critical for understanding the epidemiology of these viruses and preventing potential outbreaks. The integration of molecular characterization techniques, such as PCR and sequencing, into routine viral surveillance programs will help identify circulating strains and track their genetic evolution [32]. Moreover, environmental monitoring, including the screening of sewage and water systems for HAdV, can provide early warning of potential outbreaks and inform public health interventions [12].

In the context of Türkiye, where both viral gastroenteritis and respiratory diseases are prevalent, the inclusion of adenoviruses in routine diagnostic panels may aid in better identifying the etiological agents responsible for outbreaks [7]. Additionally, public health efforts should focus on improving sanitation, hygiene, and water treatment practices to reduce the transmission of enteric viruses, including HAdV [31].

## 5. Conclusion and recommendations

To the best of our knowledge, many reports in Türkiye cover some genotypes of HAdV-C species like C1, C2/6rc, and C5, but reports of C6 has been lacking until now. In the current study, we reported the first time isolation and characterization of an indigenous C6 strain and highlighted the persistence of its circulation in the population of Türkiye. From time to time, HAdV caused local outbreaks in some countries. Considering its geographical location, Türkiye bridges Asia and Europe. Therefore, the virus poses a threat to the country and paves the way for the entry of new HAdV strains. Therefore, our findings underscore the necessity of continuous surveillance and further analysis to investigate the prevalence, age distribution, pathogenesis, and molecular epidemiology of different HAdV genotypes. Whole genome analysis for our strain is also recommended to elucidate further the genetic structure and existence of genetic variation along with the recombination potential of circulating HAdV strains in Türkiye and to assess their public health impact.

## Supporting information

S1 File S1_raw_gel images_uncropped and unadjusted. (JPG)
