# Supplementary figures and images for: Molecular characterization of indigenous human adenovirus (HAdV) isolate from healthy infant stool sample and screening of its antibodies in archival serum samples in Türkiye
Source: PLoS One. 2025 Jul 18;20(7):e0328556. doi: 10.1371/journal.pone.0328556 (PMC12273935; doi:10.1371/journal.pone.0328556)

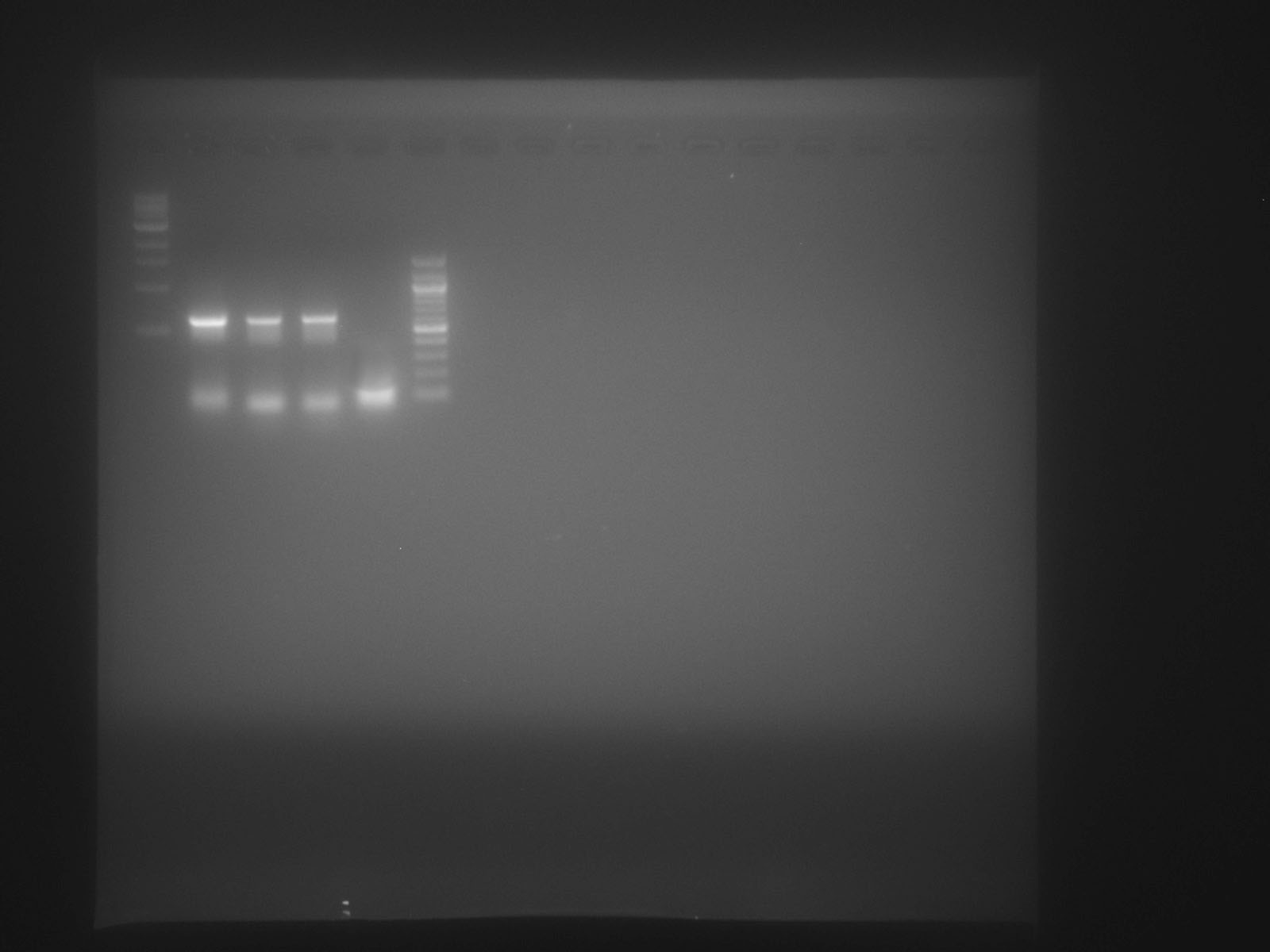

Supplement: S1 File — (JPG) [file pone.0328556.s001.jpg]
